# Molnupiravir: From Hope to Epic Fail?

**DOI:** 10.3390/v14112560

**Published:** 2022-11-19

**Authors:** Daniele Focosi

**Affiliations:** North-Western Tuscany Blood Bank, Pisa University Hospital, 56124 Pisa, Italy; daniele.focosi@gmail.com

**Keywords:** molnupiravir, MK4882, NHC, EID-1931, SARS-CoV-2, mutagenicity

## Abstract

Molnupiravir has been the first oral antiviral authorized for COVID-19 outpatients, reporting extraordinary sales and preserved in vitro efficacy against Omicron sublineages so far. However, it has recently been associated with very poor clinical efficacy, the risk of creating novel SARS-CoV-2 variants of concern, and long-term risk for mutagenicity in humans. The latter two are severe concerns, especially in the indicated population, i.e., long-replicating, immunodeficient patients. We conclude that, at this point, alternative antivirals should be preferred over molnupiravir.

## 1. What Is Molnupiravir?

Molnupiravir (developed by Merck & Co. (Hunterdon, NJ, USA; known as MSD outside the USA and Canada) and Ridgeback Biotherapeutics LP, Miami, FL, USA) is a tautomeric nucleoside analog (a.k.a. MK4882) and is the prodrug of β-d-N^4^-hydroxycytidine (NHC) (a.k.a. EIDD-1931). NHC increases G→A and C→U transition mutations [1] in replicating RNA viruses via the template strand [2,3], creating the so-called “lethal mutagenesis” (a.k.a. “error catastrophe”) [4,5]. This is different from chain termination induced by other nucleoside analogs such as remdesivir or those used for HIV and HCV treatment. Molnupiravir was originally designed as an influenza treatment [6] and became the first oral antiviral authorized during the COVID-19 pandemic by several regulatory authorities across the globe, further being recommended by WHO and scientific societies, and generating very high revenues in a short time.

With an intracellular half-life of EIDD-1931 as short as 3 h in human cell lines, it has to be taken twice a day [6]. By targeting a conserved viral gene such as the SARS-CoV-2 RNA-dependent RNA polymerase, viral resistance has been exceedingly rare so far: low-level NHC resistance was difficult to achieve and was associated with multiple transition mutations across the genome in two related coronaviruses (murine hepatitis virus (MHV) and MERS-CoV) [7]. Accordingly, molnupiravir has preserved in vitro efficacy against all recent SARS-CoV-2 Omicron sublineages [8]. However, concerns soon mounted on clinical efficacy and safety.

## 2. Poor to No Clinical Efficacy

The MOVe-OUT placebo-controlled randomized controlled trial (RCT) of molnupiravir in COVID-19 outpatients was stopped early at the recommendation of the Data Safety and Monitoring Board after approximately 50% of the sample had been recruited (May–Sep 2021, with 18.3% being convalescents as defined by the occurrence of anti-N antibodies) [9]. At the point of discontinuing the RCT, approximately 90% of the planned sample had been recruited and had available follow-up data accessible. The article was out on NEJM on 16 December 2021. Thorlund et al. noted that treatment effects reverse when examining only the post-interim analysis population (Sep–Nov 2021, with 21.7% being convalescents as defined by having anti-N antibodies: molnupiravir increasing hospitalizations by 35%) and are substantially attenuated when examining the full data [10,11]. Molnupiravir showed no overall benefit over a placebo for the resolution of COVID-19-related signs and symptoms [12]. Effectively changing the prespecified stopping time based on favorable early results is a form of p-hacking [13]. Amazingly, among those with previous infection or diabetes, the point estimate for the difference favored placebo [12]. Again, apart from severe heart failure, all the most severe risk factors were overrepresented in the placebo group, which was most evident in the interim analysis (e.g., >3-fold difference in the prevalence of chronic obstructive pulmonary diseases) [12,14]: accordingly, the hospitalization rate in the placebo group was extraordinarily high in the interim analysis (14.1% compared to 3–7% for other outpatient mAb RCTs and 4.7% in part 2 of MOVe-OUT). The study protocol had prespecified that a lost-to-follow-up patient should be considered a primary event (i.e., a hospitalization), and there was one such event in the placebo arm that conditioned statistical significance. Amazingly, one less hospitalization in the placebo group would have rendered the overall molnupiravir benefit statistically non-significant [10]. That single, untraced patient has led to signing contracts for tens of billions of USD. Post-hoc analysis of MOVe-OUT has not shown additional convincing benefits (day-29 acute care visits 6.6% vs. 10.6%; time to discharge after hospitalization being 9 vs. 12 days) [15].

There here have been at least 3 other molnupiravir trials that have been completed in similar populations but not published in peer-reviewed journals. The MOVe-IN trial, evaluating molnupiravir in hospitalized patients, showed no clinical benefits and was stopped by the trial ethics committee after slightly higher death rates were seen in the molnupiravir arm [16,17]. The Indian Central Drugs Standard Control Organization went on to approve 12 molnupiravir trials, two of which were stopped early: the ones conducted by Indian pharmaceutical companies, Aurobindo (CTRI/2021/07/034588) and MSN Laboratories Pvt Ltd., enrolled more than 2000 patients with moderate COVID-19 but showed no significant clinical benefits, according to the Indian regulatory authorities, and were discontinued [18]. The preprint from the Aurobindo RCT on 1220 patients went out on 24 February 2022: despite no patient being hospitalized in any arm, molnupiravir treatment was associated with faster clinical improvement and viral clearance [19]. In India, the Council of Medical Research left molnupiravir off its January 2022 list of recommended COVID-19 treatments [20].

Merck supported molnupiravir with the finding of another RCT run in India at record speed with the generic drug: Hetero recruited 741 patients and completed follow-up and data analysis in just 7 weeks, reporting no severe adverse events [21] (versus 116 in MOVe-OUT).

Molnupiravir as early treatment for outpatients was re-evaluated in the 2022 UK PANORAMIC RCT (ISRCTN30448031), coordinated by Oxford University, hence recruiting vaccinated patients during the Omicron wave. In PANORAMIC, the original sample size calculation of 10,600 patients assumed a 3% rate of hospital admission or death for the standard of care and 2% for patients taking molnupiravir. By 27 April 2022, PANORAMIC had enrolled 25,783 patients at 65 sites, over double the original estimate, without being halted by ethical concerns. As noted by Hill, this suggests that rates of hospital admission or death could be even lower than originally predicted [22]. Accordingly, on 4 October 2022, a preprint of the PANORAMIC RCT was posted, which found that molnupiravir did not reduce already low hospitalizations/deaths among higher-risk, vaccinated adults with COVID-19 in the community (0.8% in both placebo and intervention arms) [23].

Merck continues to investigate molnupiravir in the MOVe-AHEAD RCT for post-exposure prophylaxis in 1376 patients (NCT04939428).

## 3. Risk for SARS-CoV-2 Variant of Concern (VOC) Generation

Cytosine metabolism is known to drive the evolution of SARS-CoV-2 [24]. Merck claimed no viable SARS-CoV-2 remained after the 5-day treatment in the phase 2 trials on subjects at standard risk, 89% of whom cleared the virus anyway [25], but no virus isolation was attempted during the phase 3 trial in subjects at higher risk for disease progression (including the immunosuppressed) [12]. Concerns about ongoing replication in the majority of molnupiravir-treated patients who did not eradicate the virus after molnupiravir was first raised by William A. Haseltine (a Harvard scientist renowned as an HIV pioneer and human genome sequencer) [26,27], Carl T. Bergstrom, and James E.K. Hildreth (an FDA advisor within the Antimicrobial Drugs Advisory Committee who voted against molnupiravir EUA) on 11 November 2021. Normally, the transitions-to-transversions rate is about 2:1 for SARS-CoV-2 [28], while molnupiravir typically induces a 14:1 ratio [29]. In chronically infected patients, >80% of nucleotide changes are nonsynonymous as a result of strong selective immune pressure on the encoded antigens (with Spike overrepresented). On 5 October 2022, Ryan Hisner, a science teacher from Indiana active on GitHub as a SARS-CoV-2 variant seeker, first reported on Twitter that he had observed in GISAID 4 hypermutated SARS-CoV-2 sequences (BA.5.2.1, BA.2.3, BA.1.1, BA.4.1.1 [30]) with hallmarks of molnupiravir action (transition-to-transversion rates > 9), i.e., with a much higher incidence of synonymous transitions homogeneously distributed across the entire genome, mostly from countries with large molnupiravir use (despite after authorization black market has been widespread in low-and-middle-income countries [31]). On 3 November, a BM.2 sublineage with a transition-to-transversion ratio of 41 was reported from 9 Australian sequences [32], first suggesting that on a large scale, apparently unfit mutations can occasionally be fit. No large-scale surveillance study has been conducted to date, nor for patients formerly treated for influenza [6]. Alteri et al. recently reported that SARS-CoV-2 strains in 8 patients under molnupiravir pressure were characterized by a 6-fold higher genetic diversity compared to 7 patients under Paxlovid^®^ (Pfizer, New York, NY, USA) pressure and 5 patients under no antiviral pressure, with a peak between day 2 and day 5 [33].

## 4. Risk for Human Mutagenicity

Nucleoside analogs were first developed in the 1950s as anticancer agents (e.g., gemcitabine and 5-fluorouracil) and are known to be incorporated in both nuclear and mitochondrial human DNA [34]. Some nucleoside analogs have been withdrawn for mutagenesis on mitochondrial DNA (e.g., fialuridine [35]).

On 2 August 2021, Zhou et al. first reported that NHC is >100-fold more mutagenic than ribavirin or favipiravir against SARS-CoV-2, with antiviral activity correlated to the level of mutagenesis in virion RNA. However, NHC also displays host mutational activity in the hypoxanthine phosphoribosyltransferase (HPRT) mutation assay in Chinese hamster ovary (CHO-K1) cells, consistent with RNA and DNA precursors sharing a common intermediate of a ribonucleoside diphosphate [36]. In October 2021, molnupiravir was shown to be mutagenic in the Ames test while negative in both *Pig-a* and *cII* (Big Blue^®,^ Gentronix Ltd, Cheshire, UK; formerly Merck KGaA, Darmstadt, Germany) transgenic mouse gene reporter assays [37]. Such risks were again raised by Haseltine on 2 November 2021 [38]. In January 2022, Waters et al. reviewed the development of molnupiravir and its genotoxicity safety evaluation and concluded by advocating more thorough genotoxicity testing prior to and within phase 1 clinical trials, including error corrected next generation sequencing (duplex sequencing) studies in DNA and mitochondrial DNA of patients treated with antiviral nucleoside analogs [39]. In March 2022, Githaka, using publicly available RNA-seq data, showed that molnupiravir treatment did not increase mutations in golden hamster biopsied lung cells [40], but the study was based on just 4 vs. 2 untreated hamsters and a single body site.

In June 2022, Brandsma et al. found that molnupiravir was devoid of clear cellular toxicity, but NHC was cytotoxic and induced oxidative stress despite being not mutagenic in the ToxTracker^®^ reporter assay (Toxys, Oegstgeest, The Netherlands) based on murine embryonic stem cells [41] and in October 2022 Miranda et al. used HiFi sequencing, a technique that can detect ultralow-frequency (10^−8^ mutations per base pair) substitution mutations in whole genomes, to show that NHC increases A:T→G:C transitions in human lymphoblastoid TK6 cells at levels comparable to those observed in the plasma of humans who received clinical doses of molnupiravir [42].

Amazingly, in its only statement by the WHO Therapeutics and COVID-19 Living Guideline on molnupiravir issued on 3 March 2022, the authors concluded, based on 4796 patients enrolled in 6 RCTs (none published in peer-reviewed journals at that time) that “*The longer-term harms of molnupiravir remain unknown in the absence of clinical evidence, both for individual patients and at the population level. These include genotoxicity, emergence of resistance, and the emergence of new variants (see Mechanism of action). The conditional recommendation reflects the concern for widespread treatment with molnupiravir before more safety data become available.”* In addition, “*Certainty of evidence was rated as moderate for decreased hospitalization (rated down due to serious imprecision); low for mortality (rated down due to serious imprecision and indirectness); moderate for time to symptom resolution (rated down due to serious risk of bias); very low for mechanical ventilation (rated down due to extremely serious imprecision and serious risk of bias); and high for adverse effects leading to drug discontinuation.*” At the time of writing, i.e., one month after the release of the results of the PANORAMIC trial, nothing has been revised by WHO yet.

In the meanwhile, in a rash against caution, molnupiravir was authorized first in the UK (4 November 2021 [43]), then in the USA (23 December 2021 [44] after applying on 11 October), and in Japan (24 December 2021) [45], while on November 19 EMA advised member countries to authorize while completing its rolling review [46] (initiated on 25 October). On 22 December 2021, France, on contrary to Italy and Germany, stopped the order for its 50,000 molnupiravir doses [47,48]. Such poor efficacy (outcompeted by nirmatrelvir/ritonavir) and caveats did not prevent molnupiravir sales of USD 3.2 billion in the first quarter of 2022 (after shipping 6.4 million courses to more than 30 countries [49]) and USD 1.2 billion for in the second quarter of 2022, and to sign contracts with Sinopharm on a distribution deal in China [50]. Merck declared on 8 February 2022 that it completed manufacturing of 10 million courses of therapy (3.1 million sold to the US, 1.75 million to the UK, and 1.6 million to Japan) and remains on track to produce at least 20 million courses in 2022 to provide widespread access to molnupiravir globally [51]. At the time the PANORAMIC results were released, the UK NHS had used less than 1% of the 2.23 million doses it ordered, and it is not clear how the other 99% will be used before they pass their expiry dates [52]. The Department of Health and Social Care spokesperson said that “molnupiravir will continue to be available to high-risk patients, alongside other medicines, free testing, and vaccination, as it speeds up recovery time and reduces the amount of virus in patients” but those secondary endpoints are subjective, as was the open-label design [52].

In summary, it is amazing that one of the best-selling drugs ever was authorized for massive deployment by most regulatory authorities (including FDA and EMA) and recommended by scientific societies (including WHO) on the basis of a missed call by a single patient within the placebo arm, while ignoring unsuccessful RCTs and the inferiority to placebo in seropositive subjects (which represented the majority of the general population at the time of market launch). These approvals led to contracts for billions of USD. In addition, it is amazing that, at the same time, WHO denied recommendations for effective treatments such as convalescent plasma [53] or far cheaper repurposed drugs with largely superior efficacy and decade-long safety tracks. Clearly, this failure will create a trust crisis in the patient community, and transparency in terms of the transfer of values from Merck towards the regulatory and prescribers’ communities will be much needed to rebuild confidence.

However, what is of utmost concern is the total lack of post-marketing surveillance in terms of viral sequence mutation monitoring and secondary cancer monitoring. In particular, no clinical trial to date has systematically investigated the occurrence of viral diversification after treatment by next-generation sequencing. When it comes to secondary cancer monitoring, given that recipients have mostly been frail elderlies (ironically, many with previous underlying cancers), proving a link will be tricky. However, if this proves the case, this will likely be the greatest scandal and a major drama in the era of evidence-based medicine.

## Abbreviations

VOC: variant of concern; RCT: randomized controlled trial.
